# 341. Evaluation of Antimicrobial Use and Prescribing Patterns During the COVID-19 Pandemic in Patients Receiving Tocilizumab

**DOI:** 10.1093/ofid/ofab466.542

**Published:** 2021-12-04

**Authors:** Barbara Barsoum, Kai-Ming Chang, Nicole Mulvey, Henry Donaghy, Thien-Ly Doan

**Affiliations:** 1 Long Island Jewish Medical Center, Flushing, New York; 2 North Shore University Hospital, Manhasset, New York

## Abstract

**Background:**

Severe acute respiratory syndrome coronavirus-2 (SARS-CoV-2) infected patients experience systemic inflammation and respiratory distress, which appears to be associated with increased cytokine release. During the peak of coronavirus disease 2019 (COVID-19), tocilizumab was used to treat critically ill patients with potential cytokine storm. However, tocilizumab has an increased risk of developing serious infections.

**Methods:**

This retrospective observational chart review was approved by Institutional Review Board and evaluated patients admitted from March to November 2020, who were SARS-CoV-2 positive and received tocilizumab for the treatment group and no tocilizumab for the control group. The primary endpoint is usage of antimicrobials. The secondary endpoints are development and outcomes of secondary infections and hospital length of stay and mortality. Chi-square test was used for categorical data and Mann-Whitney test was used for continuous data.

**Results:**

A total of 160 patients were included in analysis, with 80 in each arm. 60% of patients in the treatment group required antibiotics compared to 35% in the control group (p = 0.0015), with the highest usage of anti-MRSA coverage, beta-lactams, cephalosporins, and carbapenems in both groups. Antifungal therapy was required in 21.3% of patients in the tocilizumab group compared to 6.3% in the control group (p = 0.0059), with echinocandins being the most used class in both groups. The median days of antimicrobial use in the tocilizumab group was 14 (IQR 7, 24.5) compared to 9 (IQR 6.5, 19) in the control group (p = 0.3346). In the treatment group, 60% of patients developed a secondary infection compared to 35% of patients in the control group (p < 0.0017). Secondary infection treatment failure was observed in 75% of tocilizumab patients compared to 60.7% of control patients (p = 0.1910). In hospital mortality was 50% in patients who received tocilizumab compared to 27.5% in the control group (p < 0.0039).

**Conclusion:**

Patients on tocilizumab received more antimicrobials, but with a similar spectrum of antimicrobial coverage. Patients who received tocilizumab had higher odds of developing secondary infections and expiring during their hospital stay. There were similar durations of antimicrobial therapy and treatment outcomes.

**Disclosures:**

**All Authors**: No reported disclosures

